# Diagnostic performance of 2-[^18^F]FDG PET/CT in recurrent differentiated thyroid cancer and elevated antithyroglobulin antibodies: an updated systematic review and bivariate meta-analysis

**DOI:** 10.1007/s12020-024-03989-9

**Published:** 2024-09-09

**Authors:** Domenico Albano, Arnoldo Piccardo, Alessio Rizzo, Marco Cuzzocrea, Gianluca Bottoni, Pietro Bellini, Francesco Bertagna, Giorgio Treglia

**Affiliations:** 1 Università degli Studi di Brescia, Brescia, Italy; 2 Nuclear Medicine Department, ASST Spedali Civili Brescia, Brescia, Italy; 3 Department of Nuclear Medicine, E.O. “Ospedali Galliera”, Genoa, Italy; 4 Nuclear Medicine Division, Candiolo Cancer Institute, FPO-IRCCS, Turin, Italy; 5 Division of Nuclear Medicine, Imaging Institute of Southern Switzerland, Ente Ospedaliero Cantonale, Bellinzona, Switzerland; 6 Faculty of Biomedical Sciences, Università della Svizzera italiana, Lugano, Switzerland; 7 Faculty of Biology and Medicine, University of Lausanne, Lausanne, Switzerland

**Keywords:** PET/CT, 18F-FDG, DTC, Thyroid cancer, Anti-thyroglobulin antibody

## Abstract

**Purpose:**

This updated systematic review and bivariate meta-analysis aimed to investigate the diagnostic performance of 2-[^18^F]FDG PET/CT for the detection of recurrent disease in patients with differentiated thyroid cancer (DTC) who have negative ^131^I whole body scintigraphy and increased antithyroglobulin antibodies (TgAb) levels.

**Methods:**

The current systematic review was carried out following a preset protocol, and the “Preferred Reporting Items for a Systematic Review and Meta-Analysis” served as a guideline for its development and reporting. A comprehensive research of the PubMed/MEDLINE, Embase and Cochrane library databases was conducted until June 2024.

**Results:**

Between 2002 and 2023, 13 studies (608 patients) published on this topic were selected. The pooled sensitivity, specificity, positive predictive value, negative predictive value and accuracy of 2-[^18^F]FDG PET or PET/CT were 84% (95%CI: 78−87%), 82% (95%CI: 78−86%), 72% (95%CI: 67−76%), 90% (95%CI: 87−93%) and 83% (95%CI: 79%-86%) respectively. The pooled positive and negative likelihood ratios (LR+ and LR − ) and the diagnostic odds ratio (DOR) were 0.180 (95%CI: 0.128–0.253), 3.214 (95%CI: 2.357–4.383), and 17.863 (95%CI: 10.475–30.462), respectively. No statistically significant heterogeneity among the studies was found for all the metrics evaluated (I^2^ < 50%).

**Conclusions:**

2-[^18^F]FDG PET/CT demonstrated a good diagnostic performance in patients with DTC and increased TgAb. Although more studies are warranted, the provided evidence-based data should support the integration of 2-[^18^F]FDG PET/CT in clinical and diagnostic guidelines on DTC patients with increased TgAb.

## Introduction

Differentiated thyroid carcinoma (DTC) is the most frequent endocrine cancer worldwide and is usually characterized by an excellent prognosis. Surgery (total or near-total thyroidectomy) and then radioiodine (^131^I) remnant ablation are the primary choice of treatment for these cancers. After surgery and radiometabolic therapy, ^131^I whole-body scintigraphy (WBS) and measurement of serum thyroglobulin (Tg) levels are considered the best follow-up tools [1]. Elevated Tg levels are a sensitive marker for predicting DTC recurrence or persistence of disease because thyroid parenchymal thyroid cells are the only source of Tg production [2]. After thyroidectomy with or without radioiodine ablation, persistent Tg levels can indicate residual normal thyroid tissue or metastatic disease, while undetectable Tg indicates successful ablation [3]. Stimulated Tg measurement (with recombinant human thyroid-stimulating hormone (TSH) or L-thyroxin off) is the most sensitive marker for recurrent disease, especially in patients with negative ^131^I-WBS results [4]. Nevertheless, serum levels of Tg might be affected by interference with the presence of antithyroglobulin antibodies (TgAb). For this reason, it has been recommended that Tg and AbTg levels should be measured together. Analytic interference may occur even at very low TgAb concentrations and low levels of TgAb may render Tg measurements unreliable [5–7].

Moreover, TgAb levels increase during the follow-up may be a surrogate of recurrent disease and may have a negative prognostic value especially in patients without lymphocytic thyroiditis [8–11], despite controversial positions are available in the literature.

Nowadays, fluorine-18 fluorodeoxyglucose positron emission tomography/computed tomography (2-[^18^F]FDG PET/CT) is an important imaging examination to search for the detection of recurrent disease in patients with DTC who have negative ^131^I-WBS and increased Tg and/or increased AbTg levels [12–18].

This updated systematic review and bivariate meta-analysis aims to investigate the diagnostic value of 2-[^18^F]FDG PET/CT in defining the recurrence of disease in patients with DTC who have increased AbTg and negative ^131^I WBS.

## Materials and methods

### Protocol

The current systematic review was carried out following a preset protocol, and the “Preferred Reporting Items for a Systematic Review and Meta-Analysis” (PRISMA 2020 statement) served as a guideline for its development and reporting [19]. The protocol was prepared and followed but not registered, as this is not mandatory according to the PRISMA protocol. In the Supplementary Table 1, we put the complete PRISMA checklist (Table S1). As a first step, a direct review query using the Population, Intervention, Comparator, and Outcomes (PICO) framework was done: “What is the diagnostic role (“outcome”) of 2-[^18^F]FDG PET or PET/CT (“intervention”) in patients with differentiated thyroid carcinoma and increased TgAb (“population”) compared or not to other imaging methods (“comparator”)?”. Two investigators (D.A. and A.R.) independently performed the literature search, the study selection, the data extraction and the quality evaluation. Possible disagreements were solved through a joint discussion between the two researchers.

### Search strategy

A comprehensive literature search of the PubMed/MEDLINE, Embase and Cochrane library databases was conducted together with a research on the ClinicalTrials.gov database for ongoing investigations (access date: 1 June 2024). We have created and used a search algorithm based on a combination of the terms: (a) “differentiated thyroid carcinoma” OR “DTC” AND (b) “positron emission tomography” OR “PET” AND (c) “FDG” OR “fluorodeoxyglucose”. No beginning date limit was used; the search was updated until 30 June 2024. In order to grow our research, the retrieved articles’ references were also evaluated for searching other records related to the argument of interest.

### Study selection

Original articles or subsets in studies focused on the role of 2-[^18^F]FDG PET or PET/CT in patients with differentiated thyroid carcinoma and increased TgAb were suitable for inclusion in this systematic review. Reasons for the exclusion were: (a) studies outside the field of interest; (b) preclinical studies; (c) not original studies such as reviews, editorials, comments, letters, conference proceedings, case reports or small case series (less than five patients). No language restrictions were applied. All the studies included in the systematic review were included in the statistical analysis (meta-analysis) if there were sufficient data to calculate the diagnostic performance of the index test and absence of possible overlap with other studies of the same group.

### Data extraction and collection

To avoid potential biases, the researchers separately gathered each of the studies and extracted data from the information in entire manuscript, figures, and tables. For each included study, we collected data concerning overall study information (first author, year of publication, country, study design, funding sources, number of included subjects, age, gender, reference standard); technical variables (type of scanner, PET tomograph, administered radiopharmaceutical dose, uptake time, image analysis characteristics) and TgAb method features (kind of method, mean value, normal value range).

Data about sensitivity, specificity, positive predictive value (PPV), negative predictive value (NPV) and accuracy of 2-[^18^F]FDG PET or PET/CT were also extracted. The main findings of the articles included in this review were described in Tables and in the “Results” section.

### Quality Assessment (risk of bias assessment)

A quality assessment of included articles was performed to analyze the risk of bias in individual studies to the review query. Four domains (patient selection, index test, reference standard, and flow and timing) were evaluated for risk of bias. At the same time, three sectors were assessed for applicability concerns (patient selection, index test, and reference standard) by using the QUADAS-2 tool [20].

### Statistical analysis

Diagnostic accuracy metrics were calculated from each included study through a per-patient-based analysis considering the following data: true positive, false positive, true negative and false negative findings. Pooled sensitivity and specificity were used as main outcome measures in the quantitative analysis, and these performances were calculated using a bivariate random-effects model. This statistical model takes into account the possible correlation between sensitivity and specificity [21]. Subsequently, the authors calculated pooled positive and negative likelihood ratios (LR+ and LR − ) and diagnostic odds ratio (DOR). Pooled outcome measures were provided with 95% confidence interval values (95%CI). A summary receiver operating characteristic (SROC) curve correlating sensitivity to specificity was also provided to summarize the diagnostic performance of the index test [21]. In case of significant statistical heterogeneity, subgroup analyses were planned, considering index test features, patient characteristics, technical aspects, and clinical scenarios. The inconsistency index (I-square or I^2^ index) was used to assess the presence of statistical heterogeneity (with significant heterogeneity present for I^2^ values > 50%) [21]. For the statistical analysis, we used the open-source software OpenMeta Analyst® (Brown University, Providence, RI, USA, version 10.12).

## Results

### Literature search

**Fig. 1:**
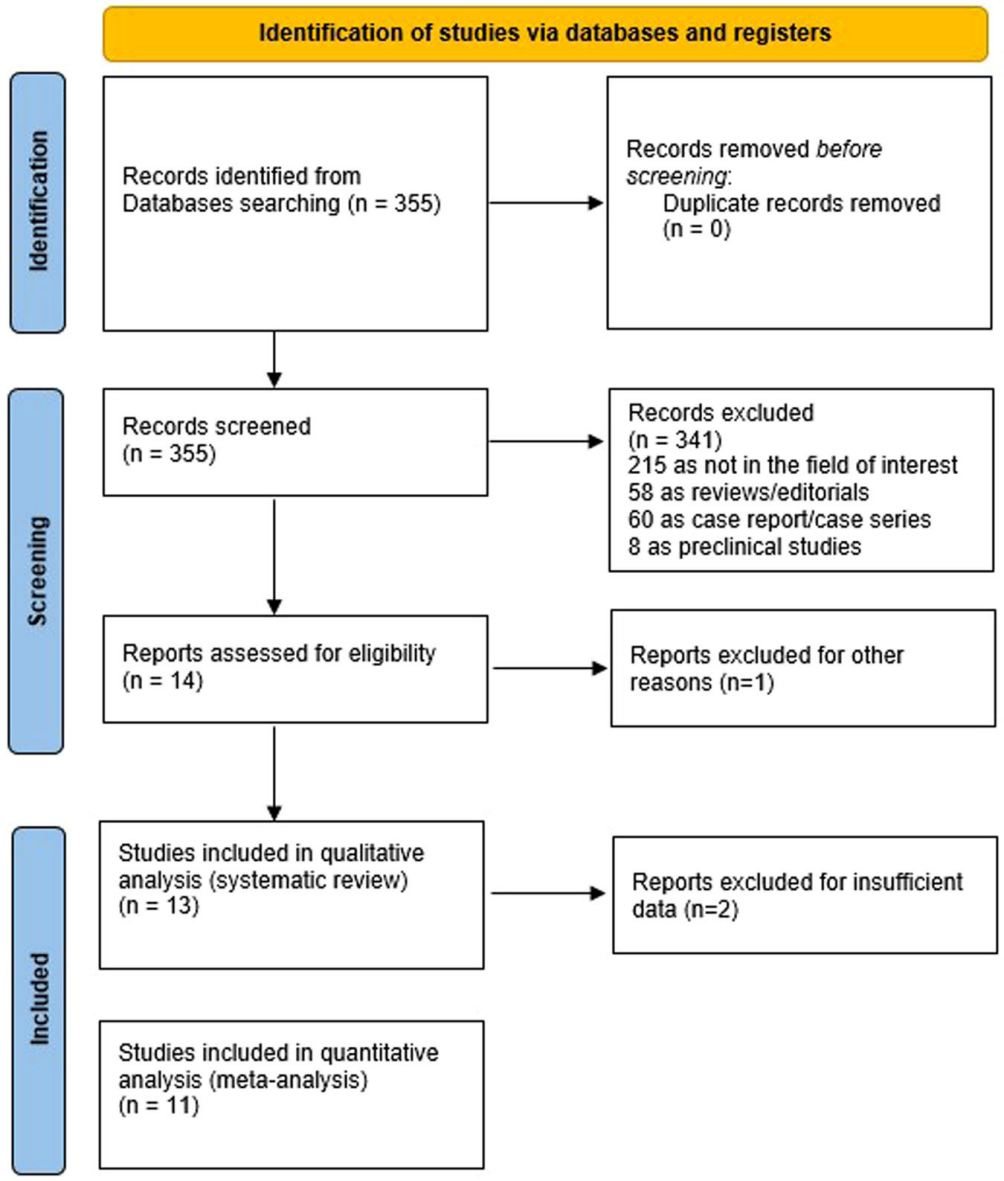
Literature search flowchart

Performing the literature search from the selected databases, we retrieved 355 records. After reading the titles and abstracts, 341 articles were excluded due to them being not in the field of interest (n = 215), small case series or case reports (n = 60), reviews or editorials (n = 58), and preclinical studies (n = 8). Lastly, 14 studies were screened in the full-text version, and 13 were included in this systematic review [6, 22–33] (Fig. 1). No additional manuscripts were added after the revision of the references of the selected records.

### Characteristics of included studies and patients

The main features of 13 articles (twelve retrospective and one prospective) included in the qualitative analysis were carefully described in Table 1. The selected articles were published between 2002 and 2023 in the Korea (3/13), Turkey (3/13), China (2/13) USA (1/13), Italy (1/13), Spain (1/13), Thailand (1/13) and Egypt (1/13). Only one research [22] disclosed financing resources in the text. Totally, 608 DTC patients with increased TgAb who performed 2-[^18^F]FDG PET or PET/CT were included. The mean age ranged from 40 to 54 years, and females were more prevalent than males in all studies except for one [23] (Table 1). The reference standard to define diagnostic performance of PET/CT was based on a combination of histopathology (when available) and clinical follow-up including radiological and biochemical findings.

**Table 1:** The main technical and clinical features

First Author	Year	Country	Study design	Funding sources	N° TgAb+ patients/total patients (%)	Age mean (range)	M:F	Reference standard
Chung JK [22]	2002	Korea	retrospective	None declared	26/26 (100%)	41.1 (17−60)	1:25	Histopathology and/or clinical follow-up
Seo JH [23]	2010	Korea	retrospective	Basic Atomic Energy Research Institute program and BK21 research program funded by the Korean Ministry of Education & Human Resources Development.	276/432 (60%)	47.8 (15−81)	58:374	Histopathology and/or clinical follow-up
Bogsrud TV [24]	2011	USA	retrospective	None declared	17/17 (100%)	51.5 (26−69)	9:8	Histopathology and/or clinical follow-up
Kingpetch K [25]	2011	Thailand	retrospective	None declared	22/22 (100%)	45 (30−62)	0:22	Histopathology and/or clinical follow-up
Viedma S [26]	2011	Spain	retrospective	None declared	7/7 (100%)	40 (23−59)	0:7	Histopathology and/or clinical follow-up
Ozkan E [27]	2012	Turkey	retrospective	None declared	31/31 (100%)	50.9 (21−82)	4:27	Histopathology and/or clinical follow-up
Ozkan E [28]	2013	Turkey	retrospective	None declared	10/59 (17%)	48.2 (19−72)	15:44	histopathology
Asa S [29]	2014	Turkey	retrospective	None declared	40/40 (100%)	43.15 (22−65)	8:32	Histopathology and/or clinical follow-up
Choi SJ [30]	2016	Korea	retrospective	None declared	9/84 (10%)	47.4	21:54^a^	Histopathology and/or clinical follow-up
Qiu ZL [31]	2017	China	retrospective	None declared	82/82 (100%)	48	32:50	Histopathology and/or clinical follow-up
Morbelli S [32]	2017	Italy	retrospective	None declared	25/71 (35%)	54 (21−83)	27:44	Histopathology and/or clinical follow-up
Liu J [33]	2018	China	retrospective	None declared	49/49 (100%)	42.7 (16−73)	6:43	histopathology
Askar HAA [34]	2023	Egypt	prospective	None declared	14/68 (20%)	41.7 (10−80)	20:48	Histopathology and/or clinical follow-up

*M* male, *F* female

^a^data available only for 75 patients

^b^median

### Characteristics of the index test

The main characteristics of the index test were summarized in the Tables 2 and 3.

**Table 2:** index test key characteristics

First Author	Hybrid imaging	Tomograph	18F-FDG mean injected activity (MBq)	Uptake time (min)	Image analysis	Semiquantitative parameters
Chung JK [22]	PET	nr	370	nr	Visual	
Seo JH [23]	PET/CT	Reveal Hirez, Siemens-CTI, Knoxville Tn USA	370−555	60	Visual and semiquantitative	SUVmax
Bogsrud TV [24]	PET & PET/CT	Advance & Discovery LS, GE Healthcare, Milwaukee, WI, USA	740	60−90	Visual	
Kingpetch K [25]	PET/CT	Biograph-16 Hi-Rez, Siemens Medical Solutions, Knoxville, TN, USA	227.5−455.4	60	Visual	
Viedma S [26]	PET & PET/CT	ECAT EXACT HR+ & biograph-16, Siemens Medical Solutions, Knoxville, TN, USA	370−434	50−60	Visual	
Ozkan E [27]	PET/CT	Biograph DUO, Biograph Truepoint; Siemens Medical Solutions, Knoxville, TN, USA	350−555	60	Visual and semiquantitative	SUVmax
Ozkan E [28]	PET/CT	Discovery; GE Healthcare, Milwaukee, WI, USA	370	60	Visual and semiquantitative	SUVmax
Asa S [29]	PET/CT	Discovery; GE Healthcare, Milwaukee, WI, USA	370	60	Visual and semiquantitative	SUVmax
Choi SJ [30]	PET/CT	Siemens, Biograph LSO HI-REZ PET/CT Illinois, USA	nr	60−90	Visual and semiquantitative	SUVmax
Qiu ZL [31]	PET/CT	Discovery STE; GE Healthcare, Milwaukee, WI, USA	370	60	Visual and semiquantitative	SUVmax
Morbelli S [32]	PET/CT	Discovery; GE Healthcare, Milwaukee, WI, USA	3−4/Kg	60	Visual	
Liu J [33]	PET/CT	Biograph 16, Siemens Medical Solutions, Knoxville TN, USA	350−450	60	Visual and semiquantitative	SUVmean
Askar HAA [34]	PET/CT	Biograph TruePoint, Siemens Healthcare, Erlangen, Germany	3.7−4.44 /Kg	60	Visual and semiquantitative	SUVmax

*PET/CT* positron emission tomography/computed tomography, *18F-FDG* 18 fluorine fluorodeoxyglucose, *SUV* standardized uptake value, *L-L SUV R* lesion to liver SUV ratio, *nr* not reported

**Table 3:** TgAb features of each study

First Author	TgAb method	Mean value IU/ml (range)	Normal value
Chung JK [22]	RIA	983.2 (25−7439)	0−100 IU/ml
Seo JH [23]	RIA	nr	0−35 IU/ml
Bogsrud TV [24]	ECLIA	416 (10−6500)	0−10 IU/ml
Kingpetch K [25]	RIA	414.6	0−200 IU/ml
Viedma S [26]	ECLIA	2486 (322.8−6500)	0−200 IU/ml
Ozkan E [27]	RIA	472.9 (27−4000)	0−40 IU/ml
Ozkan E [28]	RIA	663.4 (71.3−4000)	0−40 IU/ml
Asa S [29]	ECLIA	1101.2 (150−4000)	0−40 IU/ml
Choi SJ [30]	ELISA & ECLIA	nr	0−4.11 & 0−115 IU/ml
Qiu ZL [31]	ECLIA	479 (98−3726)	10−4000 IU/ml
Morbelli S [32]	ELISA	98 (74−616)	0−80 IU/ml
Liu J [33]	ECLIA	nr	0−115 IU/ml
Askar HAA [34]	ECLIA	nr	0−115 IU/ml

*nr* not reported, *ECLIA* electrochemiluminescence immunoassay, *ELISA* enzyme-linked immunosorbent assay, *RIA* radioimunoassay

Ten works employed PET/CT as a hybrid imaging device [22, 24, 26–33], two studies used both PET/CT and PET scanners [23, 25], and one used only PET [6]. The mean injected radiotracer activity was very heterogeneous ranging from 227.5 to 740 MBq. The time from the injection of radiopharmaceutical and acquisition was about 60 min in almost all articles. PET or PET/CT images were analyzed visually in all studies and semiquantitatively using the maximum or mean standardized uptake value (SUVmax or SUVmean) in 8 studies [22, 26–30, 32, 33].

Concerning TgAb measurements, several technical methods were used according to institutional protocol and availability: electrochemiluminescence immunoassay (ECLIA) was the most commonly used (n = 6), followed by radioimunoassay (RIA) (n = 5) and enzyme-linked immunosorbent assay (ELISA) (n = 1) and both ELISA and ECLIA (n = 1).

### Risk of bias and applicability

**Fig. 2:**
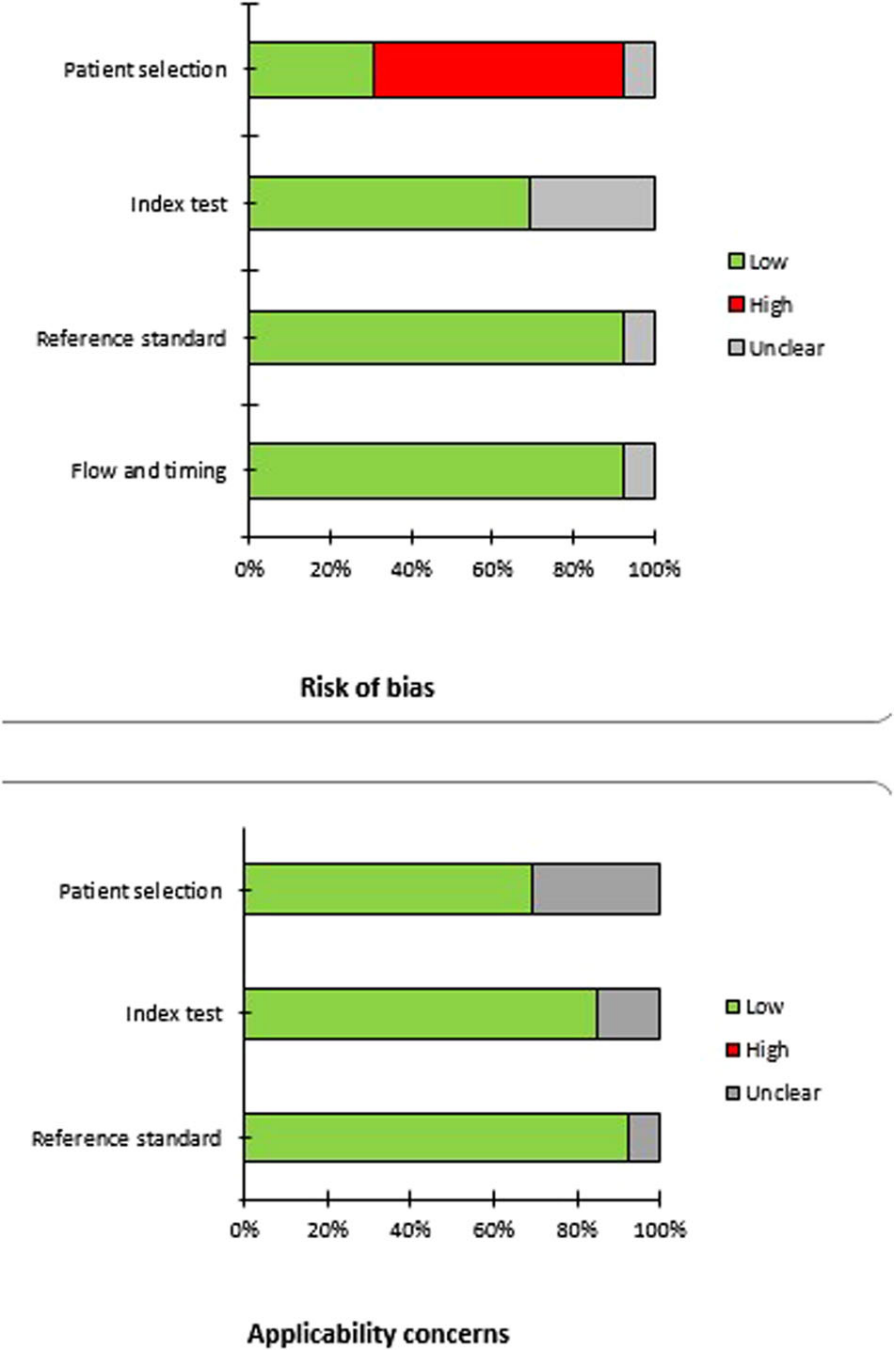
QUADAS 2 scores of the articles

The overall estimation of the risk of bias and concerns regarding the applicability of articles included in the systematic review according to QUADAS-2 are represented in Fig. 2.

### Primary results of the included studies (qualitative synthesis)

All articles investigated the diagnostic performances of 2-[^18^F]FDG PET and/or PET/CT in detecting recurrence/persistence of disease in DTC patients with increased TgAb and negative ^131^I whole body scintigraphy. Compared to conventional imaging methods (neck ultrasound and or CT), 2-[^18^F]FDG PET/CT showed a better diagnostic performance in detecting recurrence [22, 26, 27]. Liu et al. [32] investigated the combined diagnostic performance of neck ultrasound and PET/CT showing that these tools supported the clinical diagnosis and suggested subsequent treatment. Concerning the clinical impact in the patients management, only one study [32] demonstrated that PET/CT changed significantly the management in 14/49 (29%) patients. Particularly, among these 14 patients in 9 cases a re-operation was performed and in 5 a new radiometabolic therapy with high activity (Table 4).

**Table 4:** diagnostic performances reported in studies selected for meta-analysis

Author	TP	FN	FP	TN	sens	spec	PPV	NPV	accur
Chung JK [22]	11	2	1	12	85%	92%	92%	86%	88%
Seo JH [23]	28	9	32	207	76%	87%	47%	96%	85%
Bogsrud TV [24]	10	2	0	5	83%	100%	100%	71%	88%
Kingpetch K [25]	6	0	6	10	100%	63%	50%	100%	73%
Viedma S [26]	7	0	1	3	100%	75%	88%	100%	91%
Ozkan E [27]	12	2	4	13	86%	76%	75%	87%	81%
Ozkan E [28]	6	0	2	2	100%	50%	75%	100%	80%
Asa S [29]	27	7	3	3	79%	50%	90%	30%	75%
Choi SJ [30]	2	0	2	5	100%	71%	50%	100%	78%
Qiu ZL [31]	54	10	5	13	84%	72%	92%	57%	82%
Liu J [33]	14	1	10	24	93.3%	71%	58%	96%	77.5%
Combined	171	33	66	299	84%	82%	72%	90%	83%

*TP* true positive, *FN* false negative, *FP* false positive, *TN* true negative, *Sens* sensitivity, *spec* specificity, *PPV* positive predictive value, *NPV* negative predictive value, *accur* accuracy

Some included studies also explored the possible prognostic role of 2-[^18^F]FDG PET/CT, for example showing that a negative PET/CT was associated with the absence of active disease and frequently connected to a disappearance of TgAb over time [23]. On the other hand, 2-[^18^F]FDG-avid residual lesions were associated with a more aggressive disease and persistently increased TgAb [23].

Several papers proposed potential threshold values of TgAb with best accuracy to predict PET/CT findings, but these cut-off values are less reproducible due to the different TgAb assay, units of measurement and normal range. However, Kingpeth et al. [24] suggested TgAb of 414.6 UI/ml as the best value to perform PET/CT, while Qiu et al. [30] of 150 IU/ml or alternatively an increased of TgAb more than 3 years.

Concerning semiquantitative parameters, SUVmean was significantly higher in patients with increasing TgAb than decreasing (5.2 ± 1.5 vs. 2.9 ± 1.1, *p* < 0.05) [31], while SUVmax equal or greater than 4.5 may predict PET/CT findings as positive with high accuracy [24].

### Quantitative analysis (meta-analysis)

**Fig. 3:**
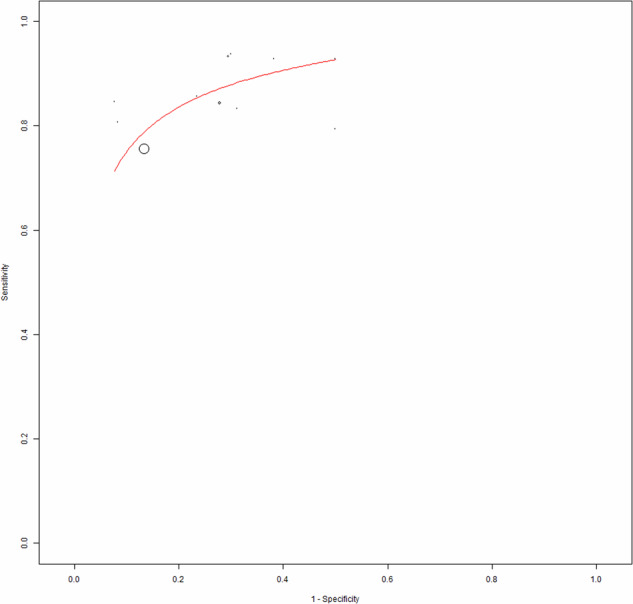
Summary ROC curve (SROC) on the performance of 2-[^18^F]FDG PET/CT in DTC patients with increased TgAb

**Fig. 4:**
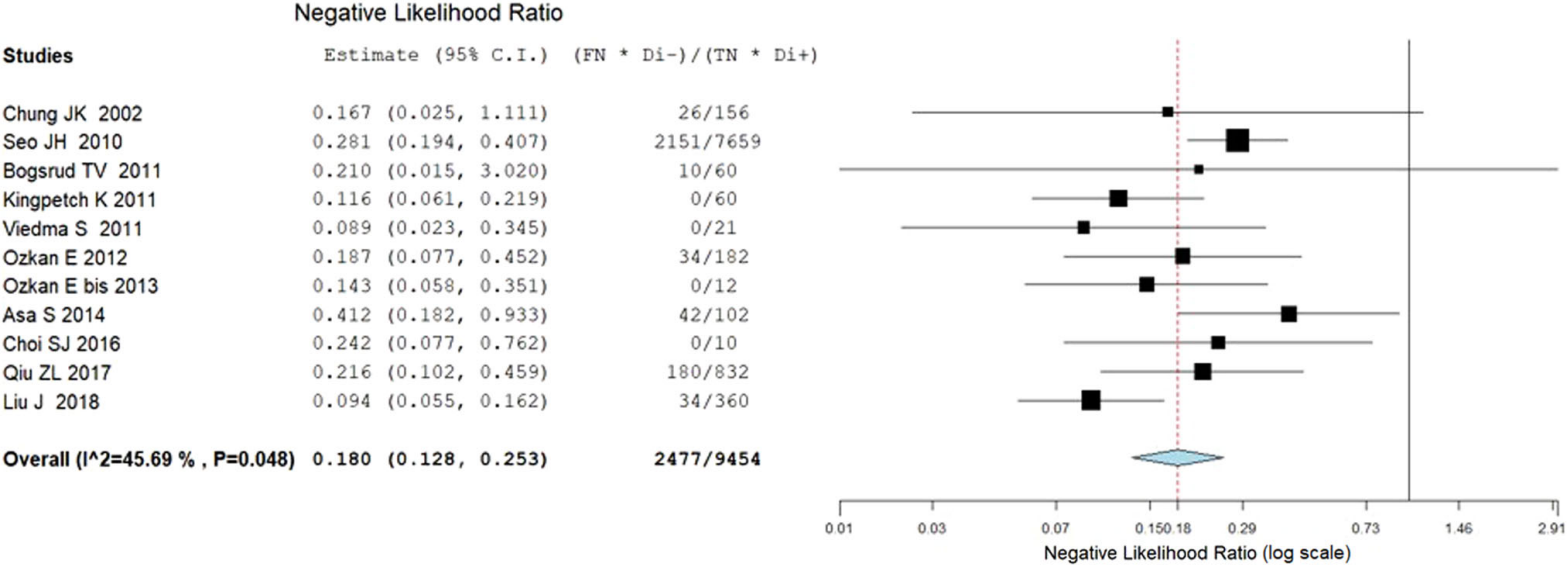
Plots of individual studies and pooled negative likelihood ratios (LR−) of 2-[^18^F]FDG PET/CT in in DTC patients with increased TgAb, including 95% confidence intervals (95% CI). The size of the squares indicates the weight of each study. The horizontal lines show the 95% CI values for each study, whereas the horizontal diameter of the rhombus shows the 95% CI value for the pooled LR−

**Fig. 5:**
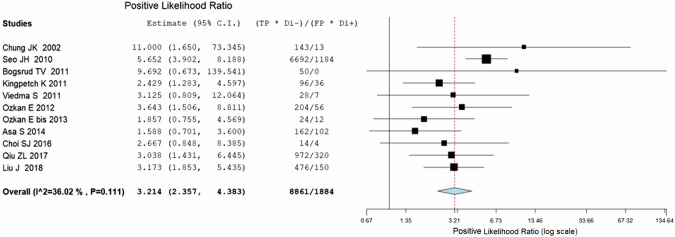
Plots of individual studies and pooled positive likelihood ratios (LR+) of 2-[^18^F]FDG PET/CT in in DTC patients with increased TgAb, including 95% confidence intervals (95% CI). The size of the squares indicates the weight of each study. The horizontal lines show the 95% CI values for each study, whereas the horizontal diameter of the rhombus shows the 95% CI value for the pooled LR+

Eleven studies including 569 patients were included for the bivariate patient-based meta-analysis [6, 22–30, 32]. The pooled sensitivity, specificity, PPV, NPV and accuracy of 2-[^18^F]FDG PET or PET/CT were 84% (95%CI: 78−87%), 82% (95%CI: 78−86%), 72% (95%CI: 67−76%), 90% (95%CI: 87−93%) and 83% (95%CI: 79%−86%) respectively. A SROC curve is represented in Fig. 3 and demonstrated a good diagnostic performance of 2-[^18^F]FDG PET/CT. The pooled LR − , LR+ and DOR were 0.180 (95%CI: 0.128–0.253), 3.214 (95%CI: 2.357–4.383), and 17.863 (95%CI: 10.475–30.462), respectively (Figs. 3, 4 and 5). No statistically significant heterogeneity amongst the studies was found for all the metrics evaluated (I^2^ < 50%).

## Discussion

**Fig. 6:**
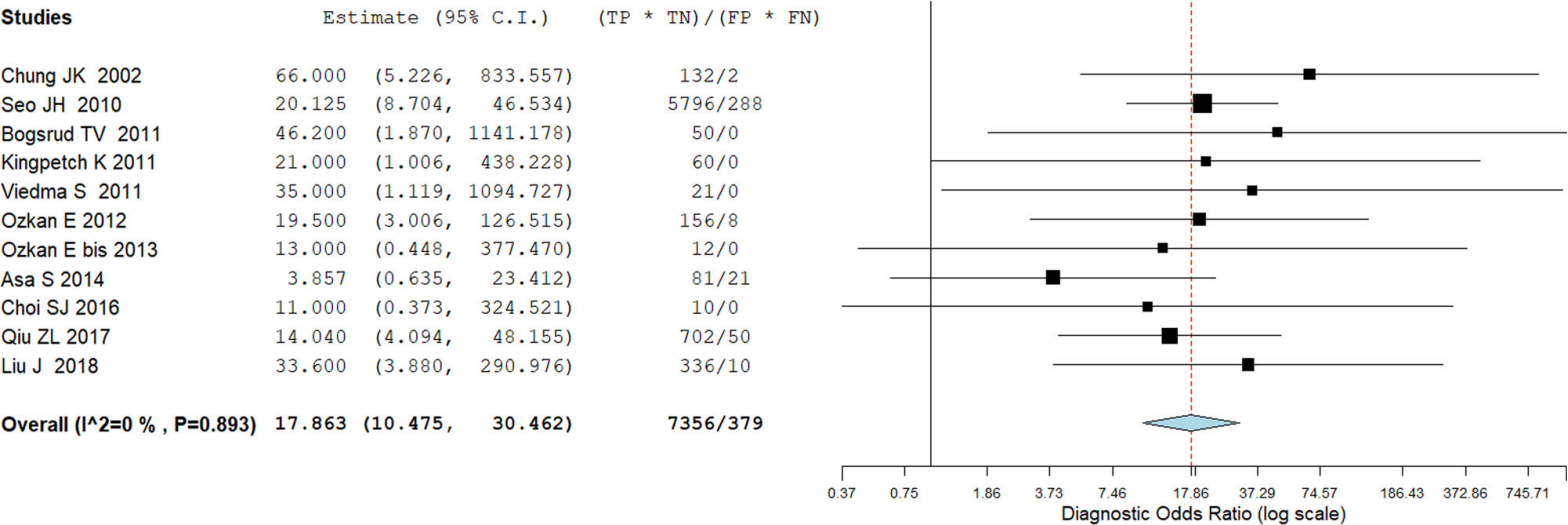
Plots of individual studies and pooled diagnostic odds ratio (DOR) of 2-[^18^F]FDG PET/CT in DTC patients with increased TgAb, including 95% confidence intervals (95% CI). The size of the squares represents the weight of each record. The horizontal lines show the 95% CI values for each study, whereas the horizontal diameter of the rhombus shows the 95% CI value for the pooled DOR

Thyroglobulin measurement is the most sensitive and important indicator of persistent and/or recurrent disease in the follow-up of DTC after total thyroidectomy and radioiodine ablation therapy. However, positive or elevated TgAb interferes with the accurate measurement of serum Tg and may mask the presence of a recurrent and/or metastatic disease. It was reported that persistently positive TgAb could be viewed as evidence of the continued presence of functional thyroid cells, either benign or malignant, and elevated TgAb might indicate the recurrent and/or metastatic disease and could be used as an alternative of the tumor marker for DTC. However, the clinical application and usefulness of TgAb for the follow-up of DTC are uncertain. Imaging studies such as the neck ultrasound and whole-body radioiodine are still used widely for the detection of the lesions. Growing evidence has shown that 2-[^18^F]FDG PET/CT is an important diagnostic tool and is able to influence the clinical management of patients affected by DTC [12]. To date, its main application concerns patients with negative post-therapeutic ^131^I -whole body scan but high Tg levels to find recurrence of disease. Other less shared indications about the role of 2-[^18^F]FDG PET/CT in DTC are the characterization of thyroid nodules suspicious for malignancy [34, 35], staging of aggressive DTC subtypes at the time of diagnosis [36] and studying patients with positive TgAb [15, 17, 18]. Elevated TgAb may indicate recurrent and/or metastatic disease and can be used as an alternative of the tumor marker for DTC [22, 37], but the real clinical significance and application for the follow-up of DTC is uncertain. Usually, lymphocytes infiltrating the thyroid tissue (typical of thyroiditis) are the main source of TgAb and they tend to disappear progressively within 3 years [38]. Thus after surgery and radiometabolic therapy with ^131^I, follow-up of DTC patients with positive TgAb may be a clinical challenge due to the impossibility to use Tg as marker. It has been demonstrated that serum TgAb levels in the first year following ^131^I ablation have an outstanding prognostic significance [39]. Durante et al. demonstrated that DTC patients with positive serum TgAb titers after primary treatment have more aggressive disease and less favorable long-term outcomes than demographically similar patients without circulating TgAb [37]. For all these reasons, identifying recurrence on patients with TgAb and negative ^131^I WBS is fundamental and needs specific alternative methods. Few studies reported the diagnostic usefulness of 2-[^18^F]FDG PET or PET/CT for the detection of recurrent and/or metastatic diseases in DTC patients with elevated serum TgAb. According to this updated systematic review and bivariate meta-analysis, 2-[^18^F]FDG PET or PET/CT has good diagnostic accuracy for the detection of recurrent and/or metastatic diseases in these patients group with a pooled sensitivity of 84% (95%CI: 78−87%), and a pooled specificity of 82% (95%CI: 78−86%). Moreover, the pooled LR − , LR+ and DOR were 0.180 (95%CI: 0.128–0.253), 3.214 (95%CI: 2.357–4.383), and 17.863 (95%CI: 10.475–30.462), respectively and no significant heterogeneity was reported for all measurements. Particularly, PET/CT performances seem to be better than conventional imaging tools like neck ultrasound and CT. This is due to the ability to derive functional information below morphological findings (Fig. 6).

Our results confirmed the data published in a previous meta-analysis [17] but are based on a larger number of studies and patients included, an updated investigation and the kind of meta-analysis performed was bivariate.

The characterization and localization of disease is essential also for the subsequent diagnostic and therapeutic management. In Liu et al. study [32], 14 patients (about a third) opted to change the treatment plan after undergoing 2-[^18^F]FDG PET/CT imaging; among them, nine patients underwent surgery again and five patients were subjected to high-dose RIT because of the multifocal transfer of lung or surgical contraindication.

A topic not well investigated is the best cut-off value for serum TgAb level that may guide the indication to perform PET/CT. Among articles included, only two [24, 30] derived potential TgAb thresholds but they were very different and directly related to the assay applied. However, in addition to the absolute value of TgAb it seems crucial to evaluate the trend over time. A downward trend seems to be less alarming and physiological, while a growing trend might be associated with a more aggressive disease and developing of new pathological thyroid cells.

The most important limitation of this systematic review and meta-analysis is the retrospective nature of most included studies. On the other hand, we did not find a significant statistical heterogeneity among the included studies, therefore, subgroup analyses to explore the heterogeneity were not performed.

Because of the relative small number of published studies and the small sample size, more studies are needed to arrive at a substantiated conclusion on the role of 2-[^18^F]FDG PET/CT in DTC patients with positive TgAb.

## Conclusions

Based on current available literature data, 2-[^18^F]FDG PET/CT demonstrated a good diagnostic performance in patients with DTC and increased TgAb. Larger prospective and multicentre studies and cost-effectiveness analyses in this field would be of great help in strengthening the role of 2-[^18^F]FDG PET/CT in this setting.

## Supplementary information

Supplementary Information
